# 

**Published:** 1893-01-21

**Authors:** 


					1893. THE hospjtal,
. CONTENTS.
tor.nj, GUI
on Friendly Workers
259
?In the Aba raot -
260
Wca? T??ncs ?I n -hZ Absract ~ - -
WIlBT^t0*y from the Earliest Times.
By E. T.
WiTHtvr' ory trom tlxe
' M.B.Oxqd.
XXXIX.-Tte Araba-Soholastio
? &Wiv?T ijl'cs ' M.B.Oxod. XXXIX.-Tte AraboSoholaBtio
Co*W?LJ?si~i?ry - ? - - - -
261
Theory and Research.-
-A New Use for
a??Stt&
"Too Pe?v Frenchman-
-Tie Use of Spermma
261
?rastenr and Jenner -
?63
UreS
264
- ?
. yaerryhinton to tlie Res 3ne?Pasteur and Jenuer
oi -unerryhn
i &,nii Missionaries
265
&?tea^alMissio
26b
*he stor Kews' ' "? ? *? ' ?' ?
LondJny A?of itlxe *nsane from Year to Year.
-City of
L?ndon ad i Insane from Year to Year.?City of
Asylum plum? Holloway Sanatorium?Killarnay District
273
Around the Hospitals
274
The Hospital cliuio ?
The Devon and Exeter Hospital.-
The Treatment o' Ohorea
267
Royal infibmary, udihbubgh^
-Un t&e Tfe&tment of Knock
Knee
Bristol Genebal Hospital.-
Treatment of Tuberculous
GIidos
269
Glamobginshibe a?d Monmouthshire Infirmary, Oabdiff.
Treatment of Acute rnenm nn
2(9
The Practitioners Bookshelf.'
-The Beginnings of Life?
The Hyciene of the fiar
270
The Institutional workshop?
Within the Hosptals.-
-TDe General Infirmary, Northampton
271
Practical departments.
-Cooking by Gas. III.
272
Soraps and Gleanings 274
Tbe Student of Medicine ? Appointments ? Vacancies.
EXTRA SUPPLEMENT.?THE NURSING MIRROR.
#ew -BiTiiA SUPflilS MiUJM
Goo/?151 Nursing1 World.?Nurses* Oo-operation?
Presol*' 01wi?niasl-London's End?A Lasting Memorial
f r m,.8 Presentations?Rest and Fresh Air?PI adin?
Nuraing?Hedicino With or Without Nursing?
_ pe?_ Pe.?> Though Kinoly?Ncrs'B by Deaconesses?Parisian
Jhe r>,_ , '8a^lsd Nurse- Short Items -
5*arni?Itiopi^ent of Children by Gymnastics. V. -
Qrautrftn Question for December -
- ^^Convalescent Hospital
CX*11<
cxxiv
cxxi*
Everybody's Opinion.?Girl Nnrtes?Oomfoitable Contribu-
tions?Endowed Bed?"Oharity Think th no Evil" .
Appointments - ? - -
_Jedico-Psychological Association
Notes and Queries ......
Wants and Workers.-Minor Appointment
Por Heading" to the Sick.?Leisure Thoughts .
Nursing- ana Nurses' Institutions in Glasgow ? - oxxvi
Where to Go -   oxxyi
cxxlr
cxxiv
? cxxv
- cxxv
- cxxv
cxxv

				

